# Review Article: Progress in fabrication of transition metal dichalcogenides heterostructure systems

**DOI:** 10.1116/1.4982736

**Published:** 2017-05-01

**Authors:** Rui Dong, Irma Kuljanishvili

**Affiliations:** Department of Physics, Saint Louis University, St. Louis, Missouri 63103

## Abstract

Transition metal dichalcogenide (TMDC) semiconductors have attracted significant attention because of their rich electronic/photonic properties and importance for fundamental research and novel device applications. These materials provide a unique opportunity to build up high quality and atomically sharp heterostructures because of the nature of weak van der Waals interlayer interactions. The variable electronic properties of TMDCs (e.g., band gap and their alignment) provide a platform for the design of novel electronic and optoelectronic devices. The integration of TMDC heterostructures into the semiconductor industry is presently hindered by limited options in reliable production methods. Many exciting properties and device architectures which have been studied to date are, in large, based on the exfoliation methods of bulk TMDC crystals. These methods are generally more difficult to consider for large scale integration processes, and hence, continued developments of different fabrication strategies are essential for further advancements in this area. In this review, the authors highlight the recent progress in the fabrication of TMDC heterostructures. The authors will review several methods most commonly used to date for controllable heterostructure formation. One of the focuses will be on TMDC heterostructures fabricated by thermal chemical vapor deposition methods which allow for the control over the resulting materials, individual layers and heterostructures. Another focus would be on the techniques for selective growth of TMDCs. The authors will discuss conventional and unconventional fabrication methods and their advantages and drawbacks and will provide some guidance for future improvements. Mask-assisted and mask-free methods will be presented, which include traditional lithographic techniques (photo- or e-beam lithography) and some unconventional methods such as the focus ion beam and the recently developed direct-write patterning approach, which are shown to be promising for the fabrication of quality TMDC heterostructures.

## I. INTRODUCTION

**Fig. 1.**
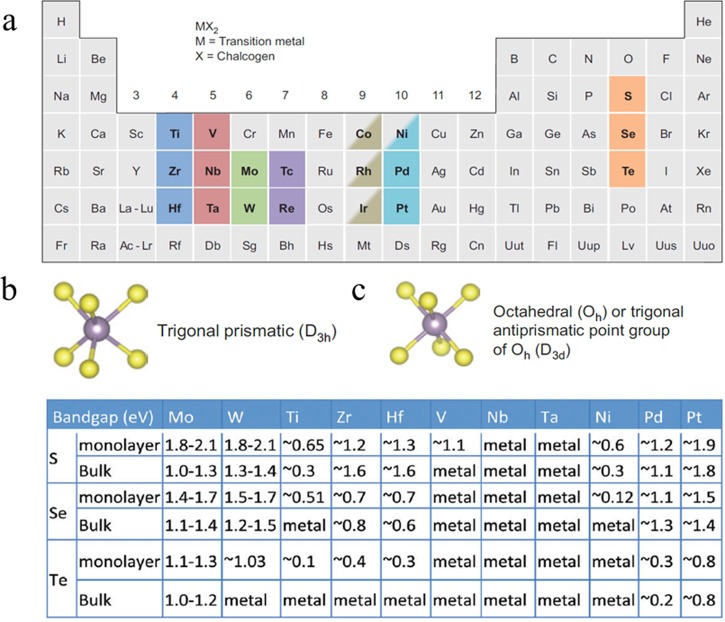
(Color online) Properties of TMDCs Semiconductor. (a) The transition metals and three chalcogen elements which are the building blocks of the 40 different TMDCs. About 40 different layered MX_2_ exist. (b) Trigonal prismatic. (c) Octahedral or trigonal antiprismatic. Table 1 The summary of common TMDCs and their band gap. (a)–(c) are reproduced with permission from Chhowalla *et al.*, Nat. Chem. **5**, 263–275 (2013). Copyright 2013 Nature Publishing Group; Table 1 in (a) is reproduced with permission from Duan *et al*., Chem. Soc. Rev. **44**, 8859–8876 (2015). Copyright 2015 Royal Society of Chemistry.

The great success of graphene research has stimulated a tremendous development in other types of two-dimensional (2D) atomic crystals.^1–3^ One of the vivid examples is a class of layered transition metal dichalcogenides (TMDCs).^4^ TMDCs have exhibited versatile and unique electrical, optical, chemical, and mechanical properties.^5,6^ TMDCs include a large family of layered materials, which can be represented by the formula MX_2_, where M is a transition metal element, and X is a chalcogen atom. Specifically, Fig. 1(a) shows the transition metals and three chalcogen elements which are the building blocks of approximately 40 different TMDCs. With the different combination of elemental makeup, the electronic properties of TMDCs can range from insulating to semiconducting and metallic, as shown in Fig. 1(a). Among these versatile properties, TMDC semiconductors provide sizable bandgaps, relatively high carrier mobility, and a high on/off current ratio in transistor switching and also have shown to be stable in air. Mechanical flexibility, optical sensitivity, and superior electronic properties make TMDC semiconductors excellent candidates for novel semiconductor systems^7–9^ and lightweight wearable and flexible applications.^10^ Jariwala *et al*.^11^ provided a review on emerging device applications of TMDC semiconductors. Radisavljevic *et al*.^7^ reviewed semiconductor transistors based on MoS_2_ single layers.

In parallel with the studies of single layered TMDCs, van der Waals (vdW) heterostructures that consist of dissimilar TMDC materials that are stacked/joined in either direction (vertical or lateral) have also been gaining extensive attention.^12–14^ In the systems of traditional semiconductor heterostructures, highly matched crystalline lattices are essential for obtaining high quality interfaces between the two (or more) dissimilar composites of building materials. Therefore, the heterostructure designs for multicompositional conventional semiconductors are complex and challenging. In contrast, the TMDCs provide a convenient opportunity to achieve high quality interfaces even in the mismatched systems. This unique opportunity is benefited from the weak vdW forces which are regarded as the dominant interactions in TMDC heterostructured systems. The recently discovered novel properties of these materials could stimulate a revolution in the design of heterostructure systems for applications such as photovoltaics, optoelectronics, spontaneous water splitting, and quantum information processing. New techniques for the design and preparation of TMDC heterostructures are still at their primary stage, and until now, several methods have been proposed and tested. More developments are still needed to further understand the influence of different fabrication methods on the resulting properties of the synthesized materials.

In this review, we highlight the recent efforts and progress in the fabrication of TMDC heterostructures. First, we will provide the readers with a brief background discussion of the properties of 2D TMDCs and their various preparation methods. Second, an in-depth review will be focused on the current progress in the fabrication of TMDC heterostructures, taking into account the challenges and the proposed solutions. Finally, a summary and outlook for the future developments in the heterostructure synthesis will also be provided.

## II. PROPERTIES OF TMDC SEMICONDUCTORS

Typically, a TMDC semiconductor single layer has a thickness of 0.6–0.7 nm, consisting of a hexagonal arrangement of transition metal atoms sandwiched between two layers of chalcogen atoms. In TMDCs, the intralayer M–X bonds between the transition metal atom and the chalcogen atoms are regarded as covalent bonds. The individual MX_2_ layers are held together by noncovalent and relatively weaker interlayer vdW forces, which allows for an easy cleavage along the surface of the individual planes/layers. The metal coordination of the TMDC semiconductor layer can be trigonal prismatic or octahedral which results from the building elements (metal and chalcogen elements), as shown in Figs. 1(b) and 1(c).

Figure 1(a) summarizes the common TMDCs and their band gap. One of the attractive features of TMDC semiconductors is the change in their electronic band structure, from the indirect band gap (bulk materials) to the direct band gap (single layer). To obtain more in-depth discussions on TMDC semiconductors and their unique physical and chemical properties, the readers can appreciate the recent reviews by Butler *et al*.^15^ Bhimanapati *et al*.,^16^ and Duan *et al*.^17^

The reported superior physical, electronic, and optoelectronic properties of TMDCs are strongly dependent on the crystallinity/quality of the MX_2_ layers and the control of the impurities in these materials and devices. Thus, the key points in integrating TMDC semiconductors into very large size circuitry is the consistency, reproducibility, and production yields of high quality TMDC layers. Moreover, reliable and scalable methods which allow for the selective fabrication of MX_2_ layers at specific locations on the substrates are required in the modern semiconductor industry. To date, various methods have been reported to produce individual and few layered TMDC semiconductor nanostructures. In the following sections (Secs. III–VII), several commonly used fabrication methods employing conventional and other less common approaches will be reviewed.

## III. PREPARATION OF TMDC SEMICONDUCTOR LAYERS

The methods for preparing TMDC semiconductor layers can be grouped into two main categories: top-down and bottom-up routes. In the top-down methods, the preparation of the MX_2_ layers is generally done from their bulk materials via exfoliation. The bottom-up methods, on the other hand, can produce MX_2_ layers on the targeted substrates from the elemental precursors. Either method has its advantages and down-sides so that the choice of a particular fabrication method largely depends on the desired amount, quality, and the production costs.

### A. Top-down method (exfoliation method)

Top-down methods can also be described as exfoliation methods which basically involve peeling off single or a few layers of materials from bulk TMDCs. This process can be done in air by mechanical exfoliation. It can also be performed chemically or electrochemically in the solution (so-called chemically or electrochemically exfoliation methods).

In a solution exfoliation method, usually small ions (e.g., Li^+^) are driven into the interlayer spacing of the bulk TMDC semiconductor in order to weaken the vdW interlayer bonding. Therefore, by further dispersion (e.g., sonication), MX_2_ layers/flakes can be peeled off and remain stable in the solution/suspensions. Moreover, doping of TMDC layers can be easily realized by adding the selected elements (dopants) during the chemical exfoliation process. Mansukhani *et al*.^18^ studied the conditions for the dispersion of MoS_2_ in aqueous solution using a range of nonionic, biocompatible block copolymers. Since the diffusion of small ions and the sonication process are, generally, not easy to control precisely, it has been therefore challenging to produce large sized monodispersed flakes and ensure perfectly uniform thicknesses of the resulting products. Moreover, the residual contaminants from the synthesis in solution may remain attached at the TMDC layers/surfaces, which could in turn degrade the TMDC quality. For more details of the recent progress in the solution based exfoliation method, the readers can appreciate the recent review article which reports on the liquid-based exfoliation.^19^

The exfoliation of TMDC atomic layers can also be performed mechanically in air by using scotch tape, i.e., so-called mechanical exfoliation method or “scotch tape” method. The mechanical exfoliation method was first developed to exfoliate single and few layer graphene from bulk graphite, and it was later extended to the fabrication of other graphenelike 2D materials.

**Fig. 2.**
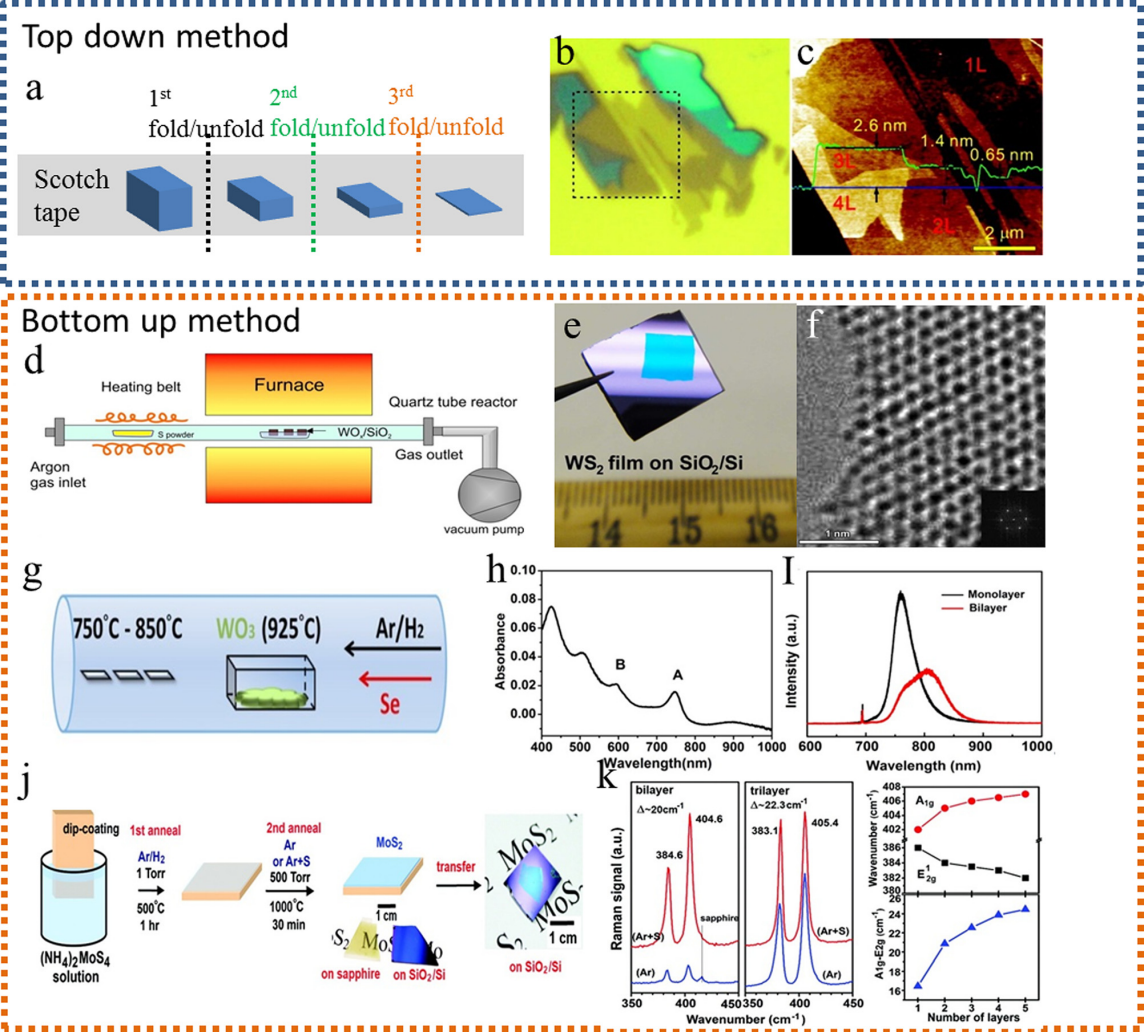
(Color online) Preparation of the TMDC semiconductor layer. (a) The schematic illustration of the mechanical exfoliation method. (b) Optical microscopy image of MoS_2_ films on the SiO_2_/Si substrate. (c) Atomic force microscopy image of MoS_2_ films for the area indicated by dotted lines in (b). (d) The schematic illustration of the single vapor method for few or single layered WS_2_ fabrication. (e) Optical image of WS_2_ films on the SiO_2_/Si substrate. (f) High resolution transmission electron micrograph of the WS_2_ single layer and edge. (g) The schematic illustration of the double vapor method for few or single layered WSe_2_ fabrication. (h) Optical absorption spectrum of the continuous WSe_2_ film. (i) Photoluminescence spectra of the WSe_2_ monolayer and bilayer with an excitation wavelength of 532 nm. (j) The schematic illustration of the thermolysis method for few layer MoS_2_ fabrication. (k) Raman spectra of bilayer and trilayer MoS_2_ films on the sapphire substrate with an excitation wavelength of 473 nm; the A_1g_ and E^1^_2g_ peak energy difference can be used to identify the number of MoS_2_ layers. (b) and (c) are reproduced with permission from Lee *et al*., ACS Nano **4**, 2695 (2010). Copyright 2010 American Chemical Society; (d)–(f) are reproduced with permission from Elías *et al*., ACS Nano **7**, 5235 (2013). Copyright 2013 American Chemical Society; (g)–(i) are reproduced with permission from Huang *et al*., ACS Nano **8**, 923 (2014). Copyright 2014 American Chemical Society; (j) and (k) are reproduced with permission from Liu *et al*., Nano Lett. **12**, 1538 (2012). Copyright 2012 American Chemical Society.

For example, the mechanical exfoliation method has been successfully used in the preparation of TMDC layers (e.g., MoS_2_, WS_2_, etc.) on the selected substrates (e.g., SiO_2_/Si, etc.).^20–22^ Figure 2(a) shows the schematic illustration of the mechanical exfoliation method for the fabrication of single and few layer TMDCs. In brief, adhesive (scotch) tapes were used to peel off MX_2_ layers from their bulk materials. By multiple folding/unfolding, the peeled MX_2_ layers can be thinned down to few or even single layers. In a similar way, as previously developed graphene transfer methods, MX_2_ layers can also be transferred onto the desired substrates for further device fabrication. For example, Lee *et al*.^23^ produced mechanically exfoliated single and few layer MoS_2_ films and measured the thickness of ∼0.6−0.7 nm which is compatible with the interlayer spacing of the bulk MoS_2_ crystals. Li *et al*.^24^ mechanically exfoliated MoS_2_ films and further fabricated field-effect transistor (FET) devices where n-type doping behavior was observed. Figure 2(b) shows the optical microscopy image of the exfoliated MoS_2_ films on the SiO_2_/Si substrate. As shown in Fig. 2(c), the thickness of MoS_2_ layers was ∼0.7 nm, which corresponds to single layers.

Because high quality MX_2_ monolayers can be produced from mechanical exfoliation, this method has been widely used in fundamental research and fabrication of a small number of individual devices for testing new design concepts. Because of its simplicity, this method has also been extensively used in the fabrication of TMDC heterostructures. Currently, the applications of the mechanical exfoliation method are limited by relatively small flake sizes, distributions in layer thicknesses, and relatively low throughput. The scalable methods which can be conveniently applied to control the MX_2_ layer thickness with good reproducibility are highly desired for practical applications

### B. Bottom-up method

With the bottom-up methods, one can directly synthesize TMDC layers on the selected substrate. At present, the chemical vapor deposition (CVD) method is one of the most widely used techniques for the synthesis of TMDCs.^25^ The CVD method can be further classified into two categories: the “single vapor” method (also called the sulfurization method) and the “double vapor” method. In the single vapor method, transition metal source-materials were usually predeposited on the substrates and consequently converted into TMDC layers through the heat treatment in a sulfur vapor environment. In the double vapor method, both transition metal sources and sulfur (or selenium) in their vapor-states were carried by inert gas flowing in the tube onto the target substrate under thermal treatment.

In addition to the CVD synthesis, other bottom-up deposition methods such as molecular beam epitaxy,^26–28^ magnetron sputtering,^29–31^ pulsed-laser deposition,^32–34^ and atomic layer deposition (ALD)^35–37^ have also been employed for the direct synthesis of TMDC layers.^38^ The bottom-up methods offer simple, relatively inexpensive, and scalable routes for the production of TMDC layers. Hence, we will focus on the CVD methods which have been often used in the fabrication of TMDC heterostructures.

#### 1. Single vapor CVD method (sulfurization method)

A schematic illustration of the single vapor method for WS_2_ fabrication is depicted in Fig. 2(d), while Figs. 2(e) and 2(f) show the optical image of WS_2_ films on the SiO_2_/Si substrate and the high resolution transmission electron micrograph (HRTEM) of the WS_2_ single layer, respectively. In this method, the transition metal source has to be predeposited on the target substrates using thermal, e-beam evaporator, sputter, or other thin film deposition tools and then transferred into the CVD system. In principle, the transition metal source can be either a pure metal or a metal oxide; however, metal oxide sources were more commonly used due to their easy oxidizing nature.

For example, Lin *et al*.^39^ evaporated MoO_3_ on top of C-face sapphire, sulfurized the oxide film at 1000 °C, and obtained wafer-scale MoS_2_ thin layers. Elías *et al*.^40^ reported the synthesis of centimeter sized WS_2_ using WO_x_ thin films as the metal source. In order to improve the WS_2_ quality, Song *et al*.^41^ sulfurized the WO_3_ film deposited by ALD because ALD has been suggested as the unique technique to control the oxide film thickness with high precision on a wafer scale. Moreover, such a sulfurization method has also been used to obtain more complicated compounds on different substrates; for example, Liu *et al*.^29^ reported the fabrication of thin Mo_1−x_W_x_S_2_ (0 ≤ x ≤ 1) ternary compounds on c-face sapphire substrates by sulfurizing the cosputtered Mo_1−x_W_x_ layers.

#### 2. Double vapor CVD method

A schematic illustration of the double vapor method for WSe_2_ layer fabrication is shown in Fig. 2(g). In brief, sulfur (or selenium) powder is placed in a boat located at the upper stream side with low temperature while transition metal oxide powder was placed in another boat in the higher temperature zone. With the flow of the carrying inert gas, both the metal oxide and the sulfur vapors were brought onto the selected substrates. As the MX_2_ layers are simultaneously formed in the process involving both vapors, this method can be regarded as an “*in situ*” deposition method that has been extensively studied. Figure 2(h) shows the optical absorption spectrum of the continuous WSe_2_ film, and Fig. 2(i) presents the photoluminescence (PL) spectra of the WSe_2_ monolayer and bilayer with an excitation wavelength of 532 nm.

Lee *et al*.^42^ prepared MoS_2_ layers using MoO_3_ powder and sulfur powder on the SiO_2_/Si substrate. Huang *et al*.^43^ prepared WSe_2_ sheets using WO_3_ powders and Se powders on sapphire substrates. Najmaei *et al*.^44^ proposed a mechanism for the MoS_2_ nucleation, growth, and grain boundary formation which is an important issue to study in order to systematically prepare large-area, single, and few layered TMDC films.

To further improve the obtained TMDC layer quality, several approaches have been proposed. For example, Ji *et al*.^45^ used a low-pressure chemical vapor deposition system to synthesis MoS_2_ on the sapphire substrate. Ling *et al*.^46^ studied the influence of seeding promoters on the growth of large sized MoS_2_ single layers and suggested that single layers can be obtained easily with the seeding promoter. Dumcenco *et al*.^47^ researched the effect of substrate quality on MoS_2_ layer growth where highly polished, epitaxial-ready grade sapphire substrates were used to control the lattice orientation. The high quality MoS_2_ layers were obtained on the centimeter scale uniformly, and the key factor was attributed to the preparation of atomically smooth sapphire terraces.

The traditional double vapor methods based on the transition metal oxide and X powder critically depend on process conditions, such as the control of the amount/location of metal oxide and X powder, the diffusion of vaporized molecules, and the flow of carrier gas. These conditions are often not easy to control because the reactants were in the form of powders. It was suggested that gas precursors (reactants) which are usually more controllable could be used as sources. In one study, Kang *et al*.^48^ reported the successful growth of MoS_2_ and WS_2_ single layered films on 4 in. silicon oxide wafers, where Mo(CO)_6_, W(CO)_6_, and (C_2_H_5_)_2_S were chosen as the precursors. In another study, Park *et al*.^49^ reported the growth of WS_2_ with a controllable layer number uniformly grown on a wafer scale using WCl_6_ and H_2_S precursors (reactants).

To summarize, the CVD process has been established as an effective and preferred method for high quality and large area synthesis of a variety of TMDC layers. Single and/or few layers have been fabricated successfully on various substrates. The selection of certain substrates (e.g., sapphire) also demonstrated the ability to enhance the preferential orientation and/or specific type of crystal formation, which provides a solution to improving material's quality. The usage of gas precursors (reactants) suggested a possible controllable route for the uniform fabrication of these materials on a wafer scale. Much work yet needs to be done to further improve these promising results until industrial standard production of TMDC materials with high yield and reproducibility can be matched. Because of the progress in the CVD, this method has also been widely employed in the fabrication of TMDC heterostructures.

#### 3. Thermolysis method

A schematic illustration of the thermolysis method for TMDC layer fabrication is presented in Fig. 2(j), while Fig. 2(k) shows the Raman spectral plots for the bilayer and trilayer MoS_2_ films on the sapphire substrate with an excitation wavelength of 473 nm. The energy difference between the A_1g_ and E^1^_2g_ peak is commonly used to identify the number of MoS_2_ layers. In the fabrication of MoS_2_ flakes, for example, ammonium tetrathiomolybdate [(NH_4_)_2_MoS_4_] powder was dissolved in a solvent to form a precursor. By dip-coating a selected substrate with the precursor and after thermal treatment, few layered MoS_2_ sheets can be obtained. Equation (1) shows the formation of MoS_2_ in the presence of H_2_ gas at high temperature

$${\left({\text{NH}}_{4}\right)}_{2}{\text{MoS}}_{4}+{\;H}_{2}\to {2\text{NH}}_{3}+{\;2H}_{2}S+{\;\text{MoS}}_{2}.$$ (1)

Since this is a solution based method with a relatively simple process,^50–52^ the authors have successfully combined the simplicity of this method with the accuracy of the selective growth process (see Sec. VII B) and developed a unique method for integrating MoS_2_ (or WS_2_) layers with graphene and TMDC heterostructures.

## IV. TMDC VERTICAL HETEROSTRUCTURES

Heterostructures have been widely used in modern semiconductor devices (e.g., p–n junctions from dissimilar materials or quantum wells by creating and engineering stacked materials with different bandgaps). TMDCs are ideal building blocks for a new type of heterostructure formation because the interlayer vdW forces allow for an easy stacking of different MX_2_ materials. Vertical TMDC heterostructures mean that different MX_2_ materials are stacked vertically layer-by-layer on top of each other. Because of the direct bandgap nature of the MX_2_ single layer, vertical TMDC heterostructures are attractive for electronic and optoelectronic applications. Novel and high performance devices based on recently developed vertical MX_2_ heterostructures have already been demonstrated. TMDC vertical heterostructures can be fabricated via either top-down methods (e.g., mechanical exfoliation) or bottom-up methods (e.g., CVD). In this section, we review the recent progress of TMDC vertical heterostructure fabrication.

### A. Mechanical method

A common method to obtain vertical heterostructures is through two or more repeating steps of mechanical exfoliation and transfer. Briefly, the first TMDC layer was exfoliated and transferred onto the target substrate followed by the second TMDC layer exfoliation and transfer. The carrier polymer can be a simple layer of commonly used polymer resist, i.e., poly(methyl methacrylate) (PMMA). For example, Chiu *et al*.^53^ fabricated the MoS_2_/WSe_2_ heterostructure as follows: the PMMA layer was first spin-coated on a MoS_2_/silicon substrate, and MoS_2_/PMMA stack was later released and mechanically transferred onto WSe_2_ flakes via substrate etching in sodium hydroxide (NaOH) solution. With a similar routine, Chiu *et al*.^54^ also prepared MoS_2_/WSe_2_ heterostructures and studied their band offsets.

This method proved to be a reliable method as Hong *et al*.^55^ reported the observation of ultrafast charge transfer in photo-excited MoS_2_/WS_2_ heterostructures, which demonstrated and confirmed their interface quality in these devices. Huo *et al*.^20,56^ micromechanically exfoliated MoS_2_ and WS_2_ flakes and demonstrated the transfer of the WS_2_ layer onto MoS_2_ flakes using PMMA coating and NaOH solution etching methods. With the obtained MoS_2_/WS_2_ heterostructures, they observed light emission quench in WS_2_ and no change in MoS_2_, which could be ascribed to the very weak interlayer coupling and inefficient charge transfer process. A water soluble polymer has also been used to increase the transferring throughput. For example, Furchi *et al*.^57^ exfoliated the bottom MoS_2_ flake onto the target substrate first and then exfoliated the top WSe_2_ flake onto a stack of polymers (carrier polymer) on a sacrificial wafer. Since the carrier polymer stack is made of the combination of water dissolvable poly(acrylic acid) (PAA) and PMMA, the PMMA/WSe_2_ flake can be transferred onto the bottom MoS_2_ flake simply by dissolving the PAA in water.

**Fig. 3.**
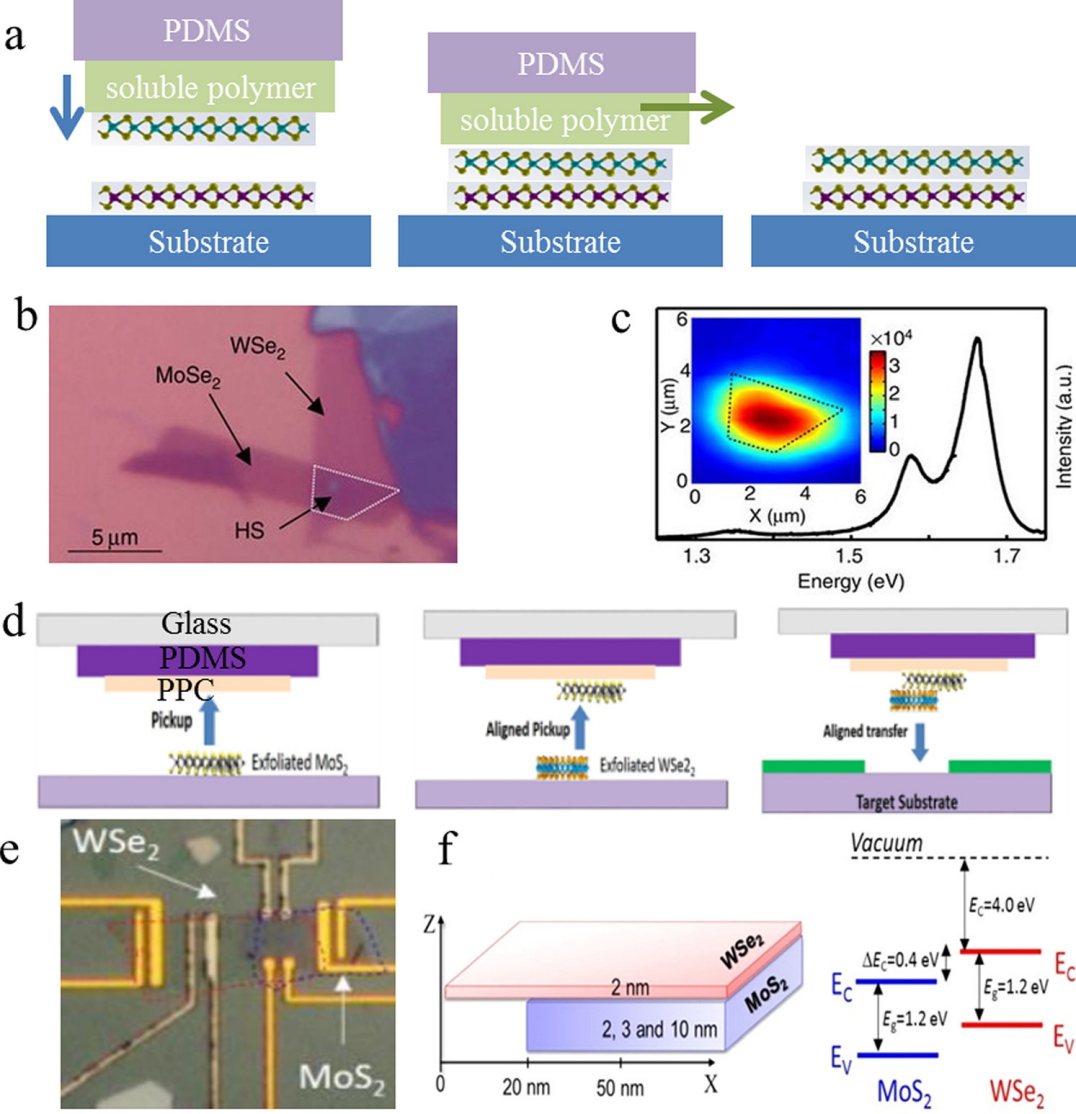
(Color online) Preparation of TMDC vertical heterostructures by the mechanical method. (a) The schematic illustration of the PDMS stamp method for TMDC vertical heterostructures fabrication. (b) Optical microscopy image of the MoSe_2_/WSe_2_ heterostructure highlighted with a dashed outline. (c) Room temperature PL of the heterostructure (the inset shows the spatial map of integrated PL intensity). (d) The schematic illustration of the dry-transfer method for MoS_2_/WSe_2_ heterostructure fabrication. (e) Optical images of MoS_2_/WSe_2_ hetero-FET. (f) The schematic illustration of MoS_2_/WSe_2_ heterostructures and the conduction band (EC) and valence band (EV) positions used for band diagram calculations. (b) and (c) are reproduced with permission from Rivera *et al*., Nat. Commun. **6**, 6242 (2015). Copyright 2015 Nature Publishing Group; (d)–(f) are reproduced with permission from Nourbakhsh *et al*., Nano Lett. **16**, 1359 (2016). Copyright 2016 American Chemical Society.

In order to have a better alignment between the second MX_2_ layer and the first one, the polydimethylsiloxane (PDMS) stamp method has been developed for the fabrication of vertical heterostructures. Figure 3(a) shows the schematic illustration of the PDMS stamp method for TMDC vertical heterostructure fabrication. Figure 3(b) shows the optical microscopy image of the MoSe_2_/WSe_2_ heterostructure highlighted with a dashed outline. The formation of the suggested heterostructures was demonstrated by the room temperature PL [Fig. 3(c), and the inset shows the spatial map of integrated PL intensity]. Tongay *et al*.^58^ reported a typical PDMS stamping method. Briefly, PDMS was spin coated directly on the WS_2_/SiO_2_/Si substrate and baked to improve the adhesion of WS_2_/PDMS. Next, the PDMS/WS_2_ sample was released from the SiO_2_/Si substrate by KOH etching and was transferred onto the MoS_2_/SiO_2_/Si substrate. Finally, the PDMS substrate was peeled off slowly from the SiO_2_/Si substrate to obtain the WS_2_/MoS_2_ heterostructure. With a similar direct PDMS stamping routine, Ceballos *et al*.^59^ fabricated the MoS_2_/MoSe_2_ heterostructure and observed indirect exciton formation in the heterostructure system. To further increase the stamping quality, water soluble polymers were inserted between stamping polymer and the MX_2_ layer so that later, the stamping polymer can be released by simply dissolving the interlayer polymer. Using the soluble polymer enhanced stamping method, Rivera *et al*.^60^ prepared MoSe_2_/WSe_2_ heterostructures and observed long-lived interlayer excitons.

Two-step mechanical exfoliation methods (dry-transfer method) have also been developed so that the entire MX_2_ heterostructure can be transferred onto the target substrate directly. Figure 3(d) shows the schematic illustration of the dry transferring method for TMDC vertical heterostructure fabrication. In the case of MoS_2_/WSe_2_ heterostructures, for example, Nourbakhsh *et al*.^61^ mechanically exfoliated WSe_2_ and MoS_2_ flakes onto two substrates, respectively. The transfer tool (glass/tape/PDMS stack) was first brought into contact with the MoS_2_ flake at room temperature. The stack was heated and cooled naturally in order to pick-up the MoS_2_ layer. In the same manner, the second flake (WSe_2_) was picked up. The obtained heterostructure (MoS_2_/WSe_2_) could then be transferred onto the target substrate. Figure 3(e) shows optical images of MoS_2_/WSe_2_ hetero-FET. Figure 3(f) shows the schematic illustration of MoS_2_/WSe_2_ heterostructures, and the conduction band (E_C_) and valence band (E_V_) positions were used for the band diagram calculations.

To summarize, the mechanical method to fabricate vertical heterostructures is laborious, and also, since supporting polymers are required for transferring, organic/polymer residues could be trapped between the MX_2_ layers and could potentially decrease the heterostructure interface quality. Moreover, similar to a single layer exfoliation routine, here, the mechanical exfoliation methods for heterostructures are also limited by the small flake size, low throughput, and significant thickness variations. Still, this stacking method has been widely reported as a method to fabricate TMDC vertical heterostructures mainly because of the following reasons. First, mechanically exfoliated TMDC layers are of higher quality, which is similar to the situation in graphene research. Also, it is convenient to prepare “four elements” heterostructures such as MoS_2_/WSe_2_ using this simple mechanical stacking, in contrast to the CVD method, in which case both the transit metal and X are different, and therefore, four different source/precursors would be required.

### B. CVD method

**Fig. 4.**
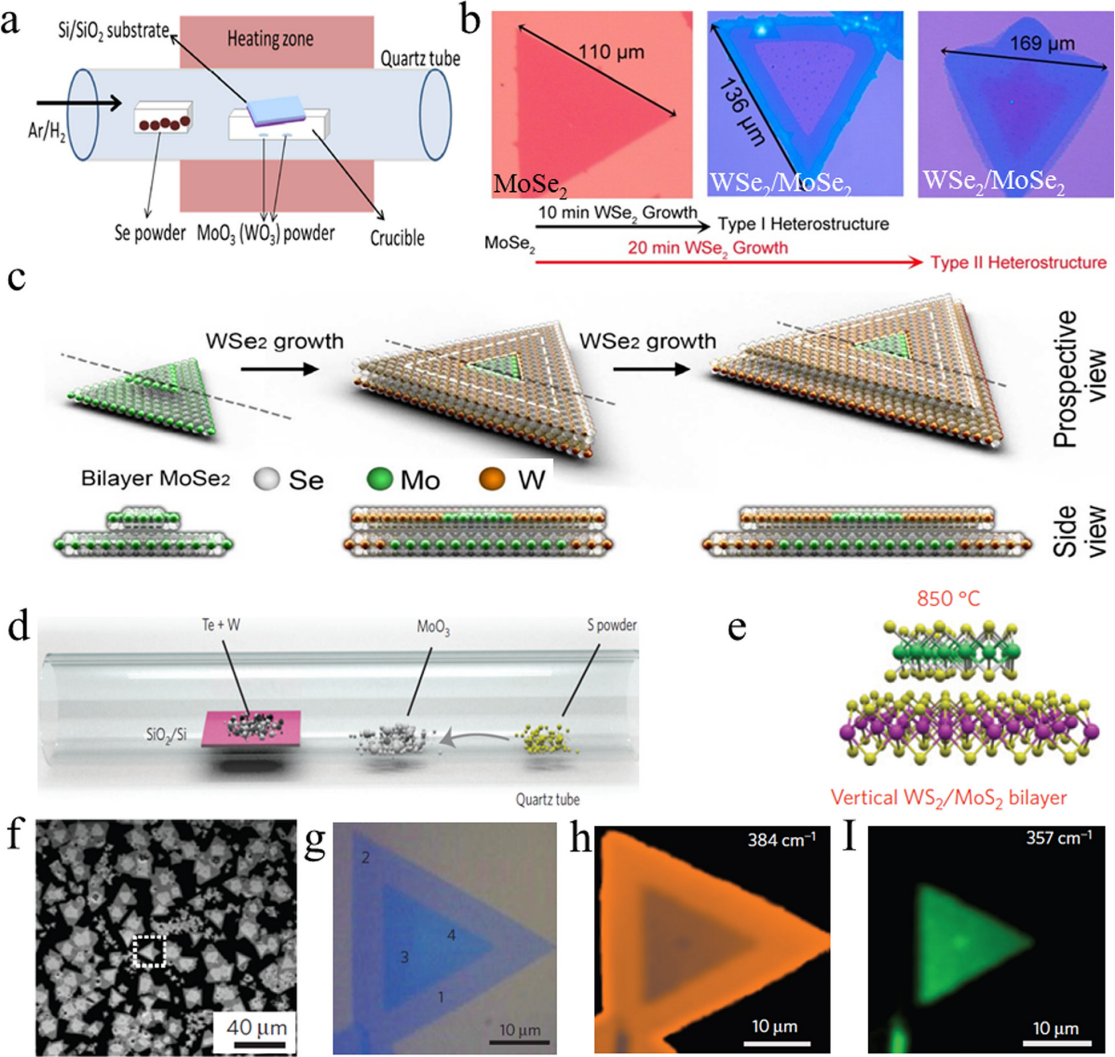
(Color online) Preparation of the TMDC vertical heterostructure via the CVD method. (a) The schematic illustration of the two-step CVD setup for WSe_2_/MoSe_2_ bilayer growth. (b) Optical images of pristine MoSe_2_, the type I WSe_2_/MoSe_2_ heterostructure, and the type II WSe_2_/MoSe_2_ heterostructure. (c) The schematic illustration of two-step growth (MoS_2_-templated WSe_2_ growth) of the WSe_2_/MoSe_2_ bilayer. (d) The schematic illustration of one-step growth of the WS_2_/MoS_2_ heterostructure. (e) Schematic of the vertical WS_2_/MoS_2_ heterostructure synthesized by the one-step method. (f) SEM image of the vertical WS_2_/MoS_2_ heterostructure. (g) Optical microscopy image of the WS_2_/MoS_2_ heterostructure used for Raman characterization. (h) Raman intensity mapping at 384 cm^−1^. (i) Raman intensity mapping at 357 cm^−1^. (a)–(c) are reproduced with permission from Gong *et al*., Nano Lett. **15**, 6135 (2015). Copyright 2015 American Chemical Society; (d)–(i) are reproduced with permission from Gong *et al*., Nat. Mater. **13**, 1135 (2014). Copyright 2014 Nature Publishing Group.

The intrinsic scalability, uniformity, and reproducibility of the CVD methods to produce single/few layered flakes provide a solution for building up the TMDC vertical heterostructures in a controllable manner. Until now, a two-step method and a one-step method have been developed for the fabrication of vertical heterostructures. Figure 4(a) shows the schematic illustration of the two-step method to prepare WSe_2_/MoSe_2_ heterostructures. Gong *et al*.^62^ first produced MoSe_2_ on the SiO_2_/Si substrate by the traditional CVD method using MoO_3_ and selenium powder as the precursors. The obtained MoSe_2_/SiO_2_/Si substrate was transferred into another CVD setup to grow the top WSe_2_ layer where WO_3_ and selenium powder were used as precursors. The thermal treatment in the second growth step would remove the absorbed small molecules (e.g., H_2_O and O_2_), and hence, no additional (thermal or chemical) treatment was needed between the first and second growth steps to eliminate adsorbents. Figure 4(b) shows the optical images of pristine MoSe_2_, type I WSe_2_/MoSe_2_ heterostructures, and type II WSe_2_/MoSe_2_ heterostructures, depending on the growth time of the WSe_2_ layer. The suggested mechanism for two-step growth (MoS_2_-templated WSe_2_ growth) of the WSe_2_/MoSe_2_ bilayer is shown in Fig. 4(c). With a very similar process, Yu *et al*.^63^ synthesized epitaxial MoS_2_/WS_2_ heterostructures. The results suggested that the transit metal source when used in the form of metal oxide (e.g., stack of MoO_3_/WO_3_) functioned the same way as that of pure metal stacks in the CVD process. This method has also been employed by Heo *et al*.^64^ to prepare “rotation-misfit-free” MoS_2_/WS_2_ (or WS_2_/MoS_2_) heteroepitaxial stacking. Gong *et al*.^65^ developed a one-step growth strategy for high-quality WS_2_/MoS_2_ vertical heterostructures by simply controlling the growth temperature. Figure 4(d) shows the schematics of the growth process for WS_2_/MoS_2_ heterostructures. MoO_3_ powder is placed in front of the target substrate (SiO_2_/Si) as the source for MoS_2_, while a mixed powder (tungsten and tellurium) is used for the WS_2_ growth. Tellurium can accelerate the melting of tungsten powder during CVD growth.

Because of the selected nucleation and growth rates, the formation of WS_2_/MoS_2_ vertically stacked bilayers was ultimately preferred at 850 °C. Figure 4(e) shows the schematic of the vertical WS_2_/MoS_2_ heterostructure synthesized by the one-step method. As seen in the scanning electron microscopy (SEM) image [Fig. 4(f)], a large amount of vertical WS_2_/MoS_2_ heterostructures was formed on the substrate. The formation of these heterostructures is also seen in optical microscopy images [Fig. 4(g)] and the Raman intensity mapping at 384 cm^−1^ [Fig. 4(h)] and at 357 cm^−1^ [Fig. 4(i)], respectively.

In addition to the traditional one-step and two-step methods, Zhang *et al*.^66^ synthesized MoS_2_/WS_2_ heterostructures using core–shell WO_3−x_/MoO_3−x_ nanowires as precursors. The nature of the core–shell WO_3−x_/MoO_3−x_ structure allowed for the controllable feeding/consumption of the oxide precursor so that MoS_2_ (and WS_2_) preferred to grow layer-by-layer.

## V. LATERAL HETEROSTRUCTURE

TMDCs have similar lattice structures with a closely matched lattice constant, and therefore, the lateral (in-plane) heterojunctions can be fabricated by seamless stitching of different MX_2_ layers. Because TMDCs have various work functions and band structures, there is an exciting opportunity and great potential for band gap engineering in different heterostructure assemblies. Moreover, lateral heterojunctions could provide an exciting opportunity to design novel devices with unique performances, for instance, utilizing exotic many body effects such as novel electron–hole condensates or superconductivity.^67^

The properties of lateral heterojunctions are determined by the nature and quality of the interface between different MX_2_, and hence, the method which can create atomically sharp interfaces is highly desired. At present, the CVD routes are the most commonly used methods for obtaining seamless lateral stitching of TMDCs.

Zhang *et al*.^68^ reported the direct synthesis of MoS_2_-WS_2_ (and MoSe_2_-WSe_2_) lateral heterostructures with aromatic molecule seeding promoters. Briefly, sulfur powders were placed upstream and heated, and WO_3-x_ vapor (evaporated from WO_3_ powders) reacted with S at the specific selected temperatures. A continuous WS_2_-MoS_2_ lateral heterostructure was obtained by the longer growth times and reduced amount of Mo.

Huang *et al*.^69^ significantly simplified the physical vapor transport method by using MX_2_ sources where the seamless quality WSe_2_-MoSe_2_ lateral heterostructure can be prepared. Briefly, the mixture of similar amounts of WSe_2_ and MoSe_2_ powders was heated in the center of the tube and was carried onto the target substrate by hydrogen. Duan *et al*.^70^ prepared WS_2_-WSe_2_ lateral heterostructures in the home-built CVD system so that the *in situ* “switch” of solid sources can be done conveniently.

**Fig. 5.**
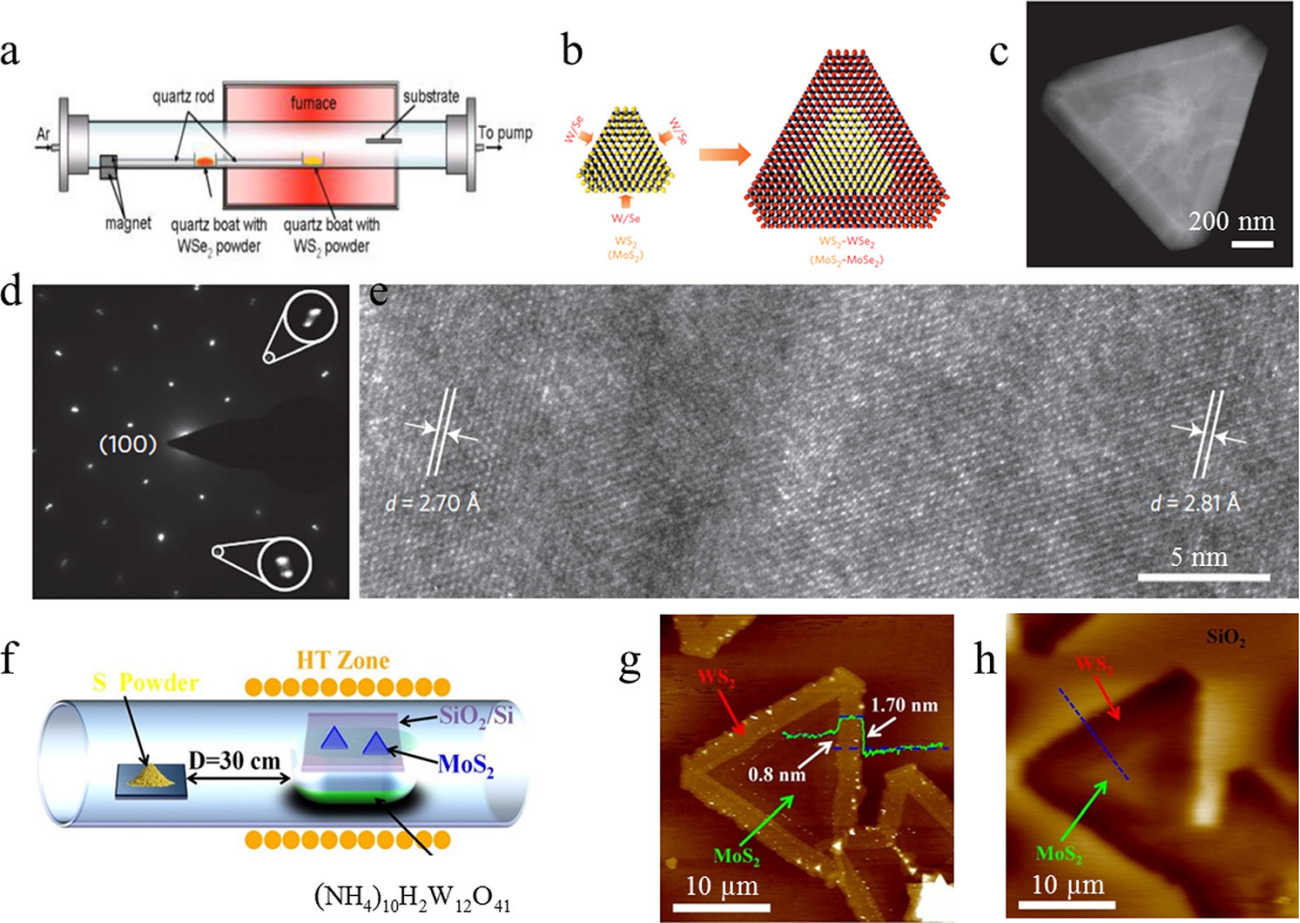
(Color online) Preparation of the TMDC lateral heterostructure via the CVD method. (a) The schematic of the one-step (*in situ*) CVD setup for the WS_2_-WSe_2_ lateral heterostructure. (b) The schematic illustration of lateral growth of WS_2_-WSe_2_ and MoS_2_-MoSe_2_ heterostructures. (c) HAADF TEM image of the heterostructure domain. (d) Electron diffraction pattern of the heterostructure interface. (e) High-resolution TEM image of the lattice fringes across the WS_2_-WSe_2_ heterostructure interface. (f) The schematic illustration of two-step growth of the MoS_2_-WS_2_ lateral heterostructure. (g) AFM image of the lateral heterostructure domain. (h) KPFM surface potential map of the heterostructure domain. (a)–(e) are reproduced with permission from Duan *et al*., Nat. Nanotechnol. **9**, 1024 (2014). Copyright 2014 Nature Publishing Group; (f)–(h) are reproduced with permission from Chen *et al*., ACS Nano **9**, 9868 (2015). Copyright 2015 American Chemical Society.

Figure 5(a) shows the schematic of the one-step (*in situ*) CVD setup for the WS_2_-WSe_2_ lateral heterostructure. Figure 5(b) shows the schematic illustration of the mechanism of lateral growth of the WS_2_-WSe_2_ and MoS_2_-MoSe_2_ heterostructures.

First, WS_2_ powder was loaded into tube furnace for the growth of WS_2_. After WS_2_ deposition, the WS_2_ powder was pushed out of the high temperature zone and WSe_2_ powder was simultaneously pushed into the hot zone. The formation of the heterostructures was further demonstrated by the high-angle annular dark-field (HAADF) transmission electron microscopy (TEM) image of the heterostructure domain [Fig. 5(c)], electron diffraction pattern of the heterostructure interface [Fig. 5(d)], and high-resolution TEM image of the lattice fringes across the WS_2_-WSe_2_ heterostructure interface [Fig. 5(e)].

Chen *et al*.^71^ reported a two-step WS_2_-MoS_2_ lateral heterostructure growth method with a simple experimental setup. Figure 5(f) shows the schematic illustration of the two-step synthesis method. First, MoS_2_ layers were synthesized on the SiO_2_/Si substrate using MoO_3_ powder as precursors. In order to prepare a lateral WS_2_ layer, in the next step, ammonium tungstate hydrate [(NH_4_)_10_W_12_O_41_·xH_2_O] was used as a reactant and sulfur powder was placed upstream of the quartz tube, resulting in WS_2_-MoS_2_ lateral heterostructure formation. As it can be observed from the atomic force microscopy (AFM) image of the lateral heterostructure domain [Fig. 5(g)] and the Kelvin probe force microscopy (KPFM) surface potential map of the heterostructure domain [Fig. 5(h)], WS_2_-MoS_2_ lateral heterostructures were fabricated successfully on the targeted substrate.

Chen *et al*.^72^ also reported a “simplified one-step method” for the formation of WS_2_-MoS_2_ lateral heterostructures where ammonium molybdate tetrahydrate (NH_4_)_6_Mo_7_O_24_·4H_2_O and ammonium tungstate hydrate (NH_4_)_10_W_12_O_41_·xH_2_O were used as Mo and W sources. Ling *et al*.^73^ reported a general synthesis method for the in-plane “parallel stitched” TMDCs with production capability.

## VI. TRILAYER HETEROSTRUCTURE

Because of the great success in the studies of the bilayer heterostructures, more recently, theoretical and experimental investigations on the trilayer TMDC heterostructures have also attracted significant attention. Lu *et al*.^74^ suggested that by intercalating a different MX_2_ single layer into the MoS_2_ bilayer, the electronic structure of the obtained trilayers could be varied. For example, the intercalation of the MoSe_2_ or WSe_2_ sheet (thus MoS_2_/MoSe_2_/MoS_2_ or MoS_2_/WSe_2_/MoS_2_ trilayer) made the “sandwich stack” system transit from an indirect-gap to a direct-gap because of the newly formed heterogeneous S/Se, while the MoS_2_/WS_2_/MoS_2_ trilayer retained its indirect-gap character, which was attributed to the lack of the S/Se interfaces.

Dataa *et al*.^75^ studied/simulated the electronic properties of the MoS_2_/MX_2_/MoS_2_ (M = Mo or W; X = S or Se) trilayer using first principles simulations. It was suggested that the bandgap depends on the inserted MX_2_ monolayer between the top and bottom MoS_2_ layers and also on their stacking configurations. Datta *et al*.^75^ also simulated the device performance of the trilayer heterostructure metal oxide semiconductor field effect transistors. Their simulations suggested that by inserting a WS_2_ monolayer between two MoS_2_ monolayers, the “ON” current can be improved.

Although many exciting properties have been reported, the lack of well controlled/reproducible and universal methods to fabricate these complex multilayer MX_2_ systems is currently limiting their developments and practical implementations. Newer techniques in fabrication and synthesis of complex heterostructure systems are required. Dong *et al*.^76^ recently have suggested a convenient method (see discussion below) to prepare MX_2_ trilayer heterostructures, i.e., the vertical trilayer structure of MoS_2_/WS_2_/MoS_2_ and the lateral tri-layer structure of MoS_2_-WS_2_-MoS_2_, which may potentially be extended to future device applications based on trilayer heterostructures.

## VII. SELECTIVE GROWTH TECHNIQUE

TMDC heterostructures, fabricated by the above described processing methods, have already been studied and proven to exhibit exciting and novel properties. Further advances in controlled selective growth, i.e., to control the shapes, geometry, and precise architecture of the formed TMDC heterostructure at predefined locations on targeted substrates, are challenging but critically needed. To date, two main approaches for the selective fabrication of TMDC heterostructures have been developed: commonly used mask-method (based on traditional lithographic techniques) and some unconventional methods to selectively grow TMDC layers and heterostructures.

### A. Conventional technique

**Fig. 6.**
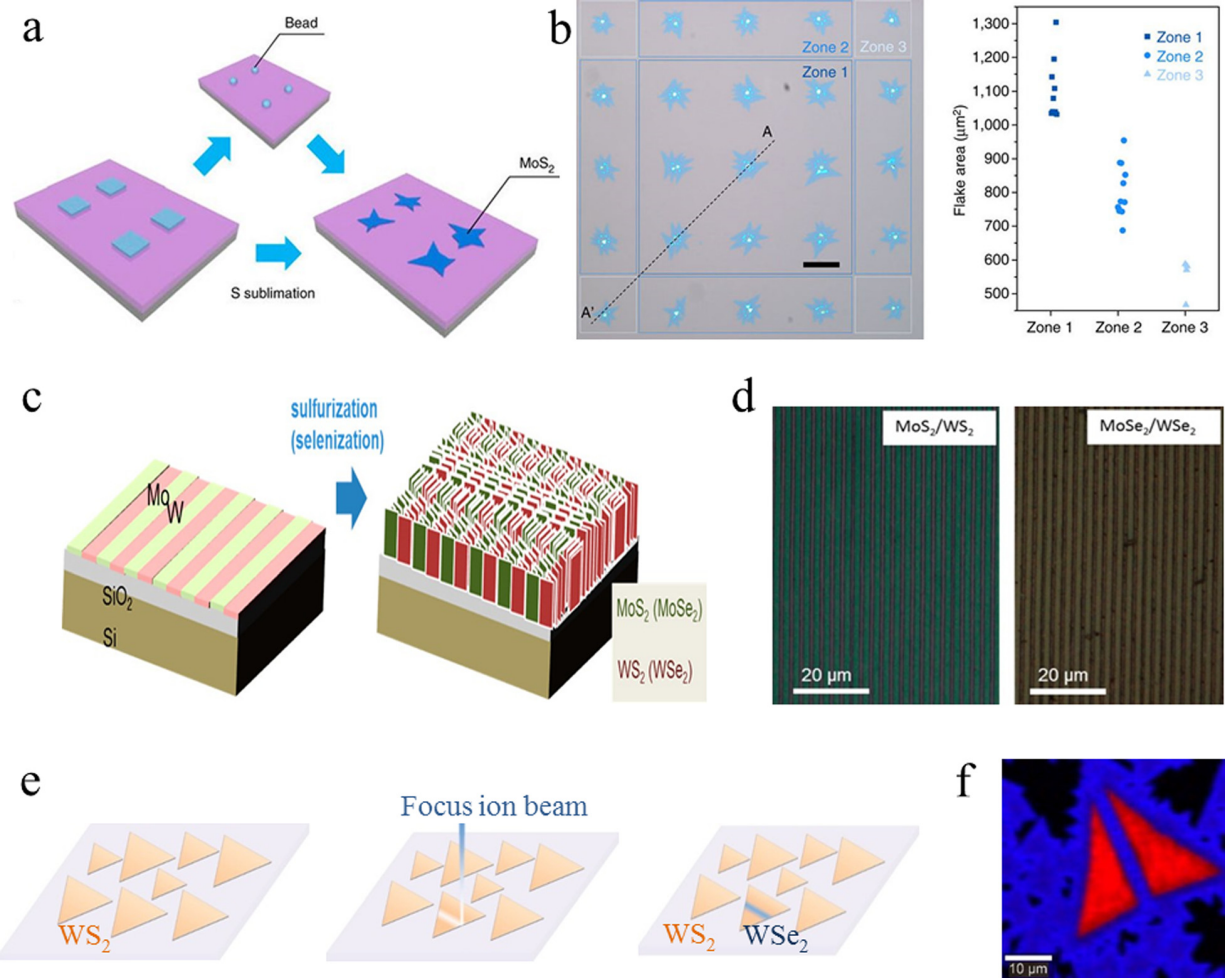
(Color online) Selective preparation of the TMDC lateral heterostructure. (a) The schematic of the MoS_2_ growth process using patterned molybdenum sources. (b) Optical image of the 5 × 5 array of the MoS_2_ monolayer grown by chemical vapor deposition (left); areas of individual flakes for zones 1 and 3 in (a). (c) The schematic illustration of Mo/W lines patterned by the photolithography technique and the formation of MoS_2_/WS_2_ and MoSe_2_/WSe_2_ heterostructures. (d) Optical images of MoS_2_/WS_2_ and MoSe_2_/WSe_2_ heterostructures. (e) The schematic illustration of the lateral TMDC heterostructures by the focused ion beam technique. (f) Raman mapping of WS_2_ crystals (triangles) and WSe_2_ flakes (large area film) surrounding the WS_2_ crystals. (a) and (b) are reproduced with permission from Han *et al*., Nat. Commun. **6**, 6128 (2015). Copyright 2015 Nature Publishing Group; (c) and (d) are reproduced with permission from Jung *et al*., ACS Nano **8**, 9550 (2014). Copyright 2014 American Chemical Society; (e) and (f) are reproduced with permission from Li *et al*., ACS Nano **10**, 10516 (2016). Copyright 2016 American Chemical Society.

In order to selectively control the position of MX_2_ layers, the commonly used lithographic techniques (e.g., photo and/or e-beam lithography) are usually employed. Han *et al*.^77^ used patterned seeds of molybdenum sources to selectively grow micrometer sized MoS_2_ flakes at specific locations on the targeted substrate. Figure 6(a) shows the schematic of the MoS_2_ growth process using patterned molybdenum sources. Briefly, a 7 × 7 array of 3 *μ*m square windows was patterned by photolithography (or electron-beam photolithography) and filled with a molybdenum containing source material. The patterned grown seeds were sulfurized in the CVD system to obtain MoS_2_ layers at preselected locations on the substrate. Figure 6(b) shows the optical images of a 5 × 5 array of MoS_2_ monolayers grown using this method.

Jung *et al*.^78^ used two-steps of photo-lithography and the metal etching procedure to fabricate MoS_2_/WS_2_ (and MoSe_2_/WSe_2_) heterostructures. Figure 6(c) shows the schematic illustration of Mo/W lines patterned using the photolithography technique and the formation of resulting MoS_2_/WS_2_ and MoSe_2_/WSe_2_ heterostructures. Briefly, the W film was first sputtered on the substrate, coated with bilayer photoresists, patterned by photomask, and selectively etched by reactive ion etching. The Mo layer was subsequently sputtered onto the exposed areas (W free areas) to obtain the periodic patterns of Mo/W lines [Fig. 6(d)]. The obtained Mo and W adjacent lines later reacted with sulfur vapor (or selenium vapor) in the CVD system to form MoS_2_/WS_2_ (or MoSe_2_/WSe_2_) heterostructures. Choudhary *et al*.^79^ also reported a lithographic protocol to prepare vertical MoS_2_/WS_2_ heterostructures where stacks of Mo/W layers were deposited and subsequently sulfurized to produce the desired heterostructures.

In addition to the common lithography techniques, other tools have also been used to fabricate TMDC heterostructures. For examples, Li *et al*.^80^ reported a photoresist-free and location-specific lithographic approach. Figure 6(e) shows the schematic illustration of the photoresist-free synthesis method. First, one MX_2_ layer was grown by a regular CVD method. Next, the obtained TMDC monolayer was patterned by a focused ion beam so that the patterned TMDC layer edges can then be used as the lateral “template” for the second MX_2_ layer. The formation of MX_2_ heterostructures was demonstrated by the Raman mapping [Fig. 6(f)] of the WS_2_ and WSe_2_ films.

### B. Direct-write fabrication method

**Fig. 7.**
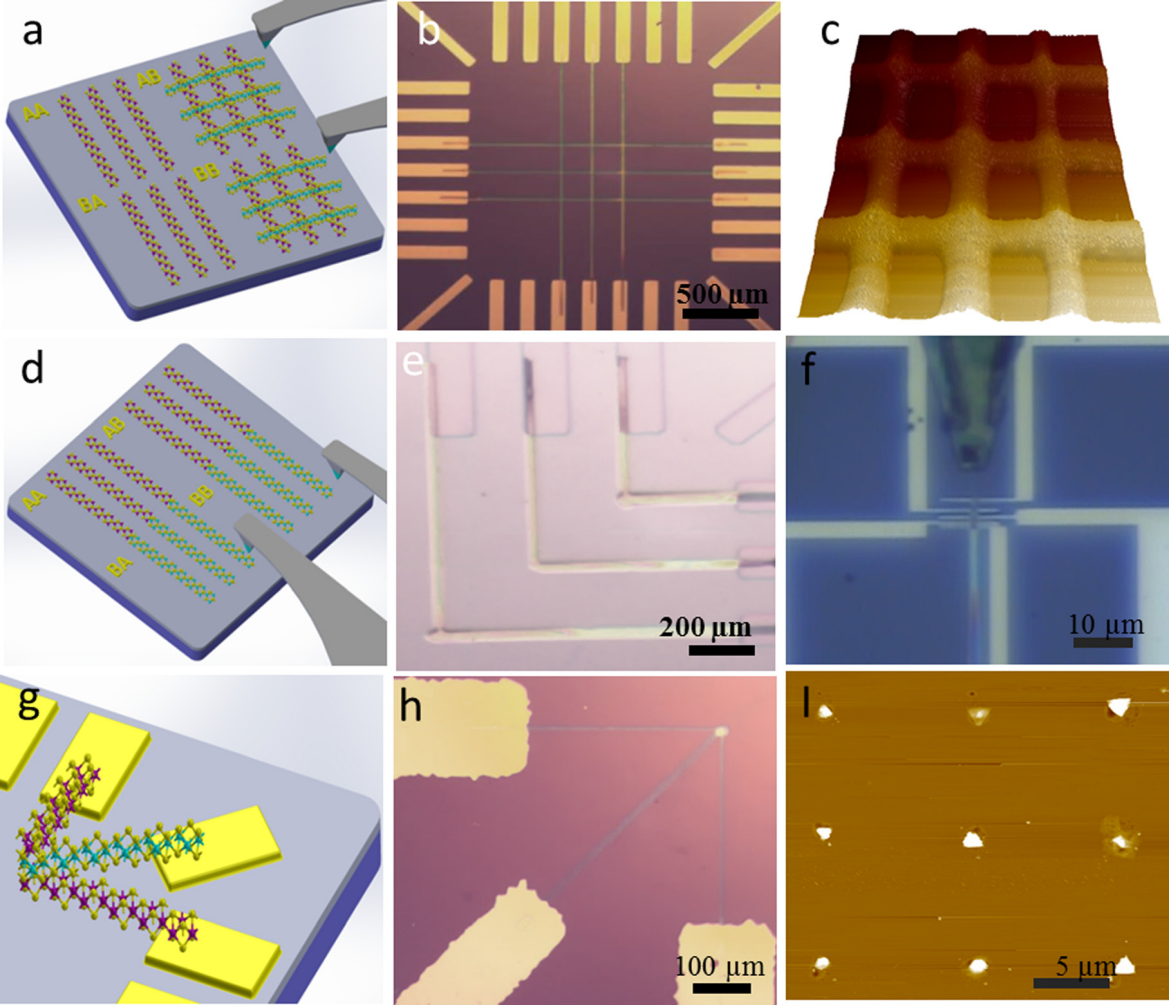
(Color online) Direct-write fabrication method. (a) The schematic illustration of the formation of TMDC vertical bilayer heterostructures. (b) Optical image of the WS_2_/MoS_2_ vertical bilayer heterostructures. (c) AFM image of the WS_2_/MoS_2_ vertical heterostructures. (d) The schematic illustration of the formation of TMDC lateral heterostructures. (e) Optical image of the WS_2_-MoS_2_ lateral bilayer heterostructures. (f) Optical image of the direct writing on devices. (g) The schematic illustration of the formation of TMDC vertical trilayer heterostructures. (h) Optical image of the MoS_2_/WS_2_/MoS_2_ vertical trilayer heterostructures. (i) AFM image of the controlled growth of WS_2_ films at preselected locations. (a)–(e), (g) and (h) are reproduced with permission from Dong *et al*., RSC Adv. **6**, 66589 (2016). Copyright 2016 Royal Society of Chemistry; (f) and (i) are the unpublished data from the authors.

The authors, Dong *et al*.,^76,81^ developed a “direct-write” patterning approach to fabricate arrays of TMDC layers and heterostructures at predefined locations on targeted substrates. Figure 7 demonstrates the concept of the direct writing process and different example architectures of TMDC heterostructures. The direct writing technique has been employed to fabricate WS_2_/MoS_2_ vertical bilayer heterostructures, as shown in Fig. 7(a) (schematic illustration), Fig. 7(b) (optical image), and Fig. 7(c) (AFM image). Additionally, Figs. 7(d) and 7(e) show the schematic illustration and the optical image of the formation of WS_2_-MoS_2_ lateral heterostructures, respectively. Figure 7(f) demonstrates the precision capabilities of the direct writing with an easy alignment of the patterned precursor to pre-existing structures (for example, prefabricated device). The principle of this technique is based on scanning probe nanolithography.^82–86^ For example, in the formation of WS_2_/MoS_2_ vertical heterostructures, two main steps are involved; the writing of W containing precursor inks with the AFM cantilever tips (multipen cantilever) on the substrate and the thermal annealing to form the first WS_2_ structure. The second step includes the subsequent writing of Mo containing precursor inks on top of the existing WS_2_ structure and subsequently the second thermal annealing process to form the MoS_2_/WS_2_ heterostructure [Figs. 7(a)–7(c)]. It is interesting to note that the second writing step with Mo containing precursor inks can be performed laterally, adjacent to the first WS_2_ layer, so that the MoS_2_-WS_2_ lateral heterostructure can also be obtained [Figs. 7(d) and 7(e)]. Furthermore, it was shown that using this convenient technique, other more complicated MX_2_ trilayer heterostructures (e.g., vertical MoS_2_/WS_2_/MoS_2_ trilayers and the lateral MoS_2_-WS_2_-MoS_2_ trilayers) could also be prepared. Figure 7(g) shows the schematic illustration of the formation of MoS_2_/WS_2_/MoS_2_ vertical trilayer heterostructures. Figure 7(h) presents the optical image of the MoS_2_/WS_2_/MoS_2_ vertical trilayer heterostructures. Since neither photo/e-beam resists nor dry/wet TMDC layer transferring was required in this mask-free process, potential polymer/organic residues at the interfaces between different MX_2_ materials can be significantly reduced improving the interface quality.

An important factor in the preparation of high quality heterostructures is the precision growth and particularly, the alignment between the dissimilar MX_2_ layers. This remains challenging for the conventional and unconventional methods of fabrication.

With further developments of more advanced direct write patterning techniques, the selective growth and alignment can be additionally improved and addressed to a broader selection of nanoscale materials and heterostructures. Recently, the controlled growth of MX_2_ flakes at preselected locations was also carried out by the authors, as shown in the AFM image of the 3 × 3 array of WS_2_ flakes [Fig. 7(i)].

To summarize, the direct write fabrication is a unique approach which allows for the formation of high quality vertical WS_2_/MoS_2_ and lateral MoS_2_-WS_2_ heterostructures with controllable lengths, widths, and thicknesses at predefined locations. This mask-free approach has also been applied to produce more complex MX_2_ trilayer heterostructures with promising results. Moreover, one can envision utilizing the cantilever chips with thousands of tips for the large scale and high throughput patterning. This unconventional scalable approach encompasses the simplicity of the solution method and the precision of scanning probe nanolithography, and therefore, it could be considered the promising technique for the large scale integration of TMDCs in device applications.

## VIII. SUMMARY AND OUTLOOK

The TMDC semiconductors have rich physical and chemical properties and therefore have become a subject of an active fundamental physics and materials research and development of novel devices. The nature of weak interlayer vdW interactions in TMDCs provides a unique avenue for the realization of high quality and atomically sharp heterostructures in different geometries. The diverse electronic properties of TMDCs have opened up new opportunities for designing, developing, and testing heterojunction systems with tunable band alignment and novel functionalities. In order to pursue the integration of TMDC heterostructures with the existing semiconductor industry, it is critically important to develop universal methods for large size controlled production of high quality TMDCs with high-throughput. To date, many realized devices and architectures are based on protocols for the exfoliation of bulk crystals (top-down method) and this process could be difficult to scale-up for the large area integration.^7,16,87,88^ It must be noted that although very high quality MX_2_ monolayers can be fabricated via mechanical exfoliation, the further development of this method is limited by the relatively small TMDC layer sizes and low yields. The chemical (or electrochemical) exfoliation methods in principle allow the large scale production of MX_2_ flakes; however, the obtained TMDC layers were usually doped with the chemical elements present in the solution, which might hinder their suitability for applications in the high performance electronics.

Furthermore, the applications of the top-down methods for the production/fabrication of high quality TMDC heterostructures encounter more challenges because of the inherent issues such as the complicated transferring processes and the common difficulty in controlling the alignment between the first MX_2_ layer and the second layer and potentially additional subsequent layers. In contrast, the bottom-up methods allow for the direct fabrications of large sized and uniform TMDC layers and on the selected substrate with a relatively high level of reproducibility and certainly in a more controllable fashion. If required (for specific applications), the amount of doping can also be better controlled during the production process. Therefore, the bottom-up methods are becoming increasingly more important as compared to the top-down approaches with respect to the fabrication of high quality and complex TMDC heterostructures with high-throughput.

Recently, increasing efforts have been devoted to the thermal CVD process employing one-step or two-step methods due to their scalability and flexibility. These CVD methods conveniently allow for precise control of the process parameters (pressure, temperature, carrier gas, and reactants), and they are well suited for selective growth of desired materials. The conventional approaches to the selective growth of the TMDCs on predefined locations are based on photo or e-beam lithography with the use of polymer/organic layers as lithographic masks. To date, a majority of lithographically patterned structures have been prepared using those conventional tools. We have also reviewed more recent unconventional methods for the preparation of high quality MX_2_ heterostructures and discussed their advantages and/or drawbacks. Along with these recently employed fabrication methods, we have additionally introduced a direct write patterning technique as a unique approach for the production of quality vertical and/or lateral MX_2_ heterostructures with controllable geometries. This approach combines the simplicity and flexibility of mask-free patterning and nanoscale precision of scanning probe lithography and enables the selective growth of different materials and hence could be promising for the integration of TMDCs into device applications. The studies of TMDC heterostructures are still in their exploratory stages; newer methods and platforms for materials designs and device fabrication should and most certainly will continue to be further investigated and developed.
